# National household survey of adverse childhood experiences and their relationship with resilience to health-harming behaviors in England

**DOI:** 10.1186/1741-7015-12-72

**Published:** 2014-05-02

**Authors:** Mark A Bellis, Karen Hughes, Nicola Leckenby, Clare Perkins, Helen Lowey

**Affiliations:** 1 Centre for Public Health, World Health Organization Collaborating Centre for Violence Prevention, Liverpool John Moores University, 15-21 Webster Street, Liverpool L3 2ET, UK; 2 Public Health Wales, Hadyn Ellis Building, Maindy Road, Cardiff CF24 4HQ, UK; 3 Department of Academic Neonatal Medicine, Chelsea and Westminster Campus, Imperial College London, Fulham Road, London SW10 9NH, UK; 4 Knowledge and Intelligence Team (North West), Public Health England, 15-21 Webster Street, Liverpool L3 2ET, UK; 5 Blackburn with Darwen Borough Council, Specialist Public Health Directorate, 6th floor, 10 Duke Street, Blackburn BB2 1DH, UK

**Keywords:** Child abuse, Childhood, Alcohol, Smoking, Violence

## Abstract

**Background:**

Epidemiological and biomedical evidence link adverse childhood experiences (ACEs) with health-harming behaviors and the development of non-communicable disease in adults. Investment in interventions to improve early life experiences requires empirical evidence on levels of childhood adversity and the proportion of HHBs potentially avoided should such adversity be addressed.

**Methods:**

A nationally representative survey of English residents aged 18 to 69 (n = 3,885) was undertaken during the period April to July 2013. Individuals were categorized according to the number of ACEs experienced. Modeling identified the proportions of HHBs (early sexual initiation, unintended teenage pregnancy, smoking, binge drinking, drug use, violence victimization, violence perpetration, incarceration, poor diet, low levels of physical exercise) independently associated with ACEs at national population levels.

**Results:**

Almost half (47%) of individuals experienced at least one of the nine ACEs. Prevalence of childhood sexual, physical, and verbal abuse was 6.3%, 14.8%, and 18.2% respectively (population-adjusted). After correcting for sociodemographics, ACE counts predicted all HHBs, e.g. (0 *versus* 4+ ACEs, adjusted odds ratios (95% confidence intervals)): smoking 3.29 (2.54 to 4.27); violence perpetration 7.71 (4.90 to 12.14); unintended teenage pregnancy 5.86 (3.93 to 8.74). Modeling suggested that 11.9% of binge drinking, 13.6% of poor diet, 22.7% of smoking, 52.0% of violence perpetration, 58.7% of heroin/crack cocaine use, and 37.6% of unintended teenage pregnancy prevalence nationally could be attributed to ACEs.

**Conclusions:**

Stable and protective childhoods are critical factors in the development of resilience to health-harming behaviors in England. Interventions to reduce ACEs are available and sustainable, with nurturing childhoods supporting the adoption of health-benefiting behaviors and ultimately the provision of positive childhood environments for future generations.

## Background

Non-communicable diseases (NCDs) have risen to become the greatest contributors to burden of disease globally, accounting for two thirds of all deaths (34.5 million [1]) and 54% of disability adjusted life years (DALYs; 1.3 billion [2]) in 2010. In high-income countries this proportion is typically much higher, reaching 87% in western Europe [3]. Two of the most common causes of NCDs, alcohol use and tobacco smoking (including second-hand smoke), are now the leading risk factors for burden of disease and injury in 15 to 49-year-olds, and globally attributed to around 800,000 and 565,000 deaths respectively in this age group in 2010 [4]. Obesity and drug use contributed an additional 325,000 and 107,000 deaths, respectively [4]. Thus, at global [5], regional (for example, Europe [6]), and national levels, tackling NCDs and the health-harming behaviors (HHBs) that cause them are health and economic priorities.

Although HHBs such as drug misuse, smoking, violence, and poor nutrition are apparent in all sectors of society, they are typically more prevalent in the poorest communities [7-9]. However, even in such communities, most individuals in high-income countries do not abuse alcohol, take illicit drugs or smoke, and their diet and exercise regimens remain sufficiently balanced to at least avoid obesity (for example, in England [10]). Equally, although often at a lower prevalence, HHBs are far from absent in more affluent communities. Consequently, although socioeconomic gradients are strong predictors of HHBs, additional factors are required to explain the resilience and susceptibility of individuals to developing HHBs throughout the life course.

Over the past two decades, studies have begun to explore how early life experiences impact on behavior and health during adolescence and adulthood. Adverse childhood experience (ACE) studies show that adult health profiles relate to the abuse individuals experienced during childhood as well as to other childhood stressors such as parental substance misuse, incarceration, and domestic violence [11,12]. The initial ACE study in the USA emerged from work identifying strong relationships between ACEs and adult obesity [13]. Since then, ACEs have been related to increased propensity for substance use (alcohol, tobacco, and drugs), anti-social behavior, and ultimately development of cardiovascular disease, cancer, chronic lung disease, and diabetes [11,12]. Critically, studies have established that the quantity of stressors (that is, the ACE count) is an important predictor of poor behavioral and consequently poor health outcomes over the life course. Moreover, exposure to multiple stressors in childhood is also associated with subsequent unintended pregnancies [14], and being a victim or perpetrator of violence, including intimate partner violence [15]. Together, these sexual and violent behaviors create a mechanism for intergenerational passage of ACEs and their health consequences [16].

Through a range of evidence-based interventions, ACEs are a modifiable factor in children’s lives [17]. However, there is currently little understanding of the potential impact of reducing ACEs independent of socioeconomic factors (for example, deprivation) that are also associated with poor health choices and anti-social behavior. Consequently, here we used a national ACE survey to measure levels of ACEs across England, calculated the prevalence of exposure to multiple ACE counts, and examined the relationships between ACE exposure and HHBs. After accounting for deprivation and other demographic effects, we modeled the impact of reduced ACE prevalence on resilience to HHBs. We considered how supportive childhoods allow individuals to resist the commercial, cultural, and environmental pressures that promote substance use, obesity, and anti-social behavior throughout the life course.

## Methods

A national household survey of adults resident in England was undertaken between April and July 2013. Ethical approval was obtained from Liverpool John Moores University and the study adhered to the Declaration of Helsinki.

The study used an established survey tool [16] that includes questions on participant demographics, ACEs, and HHBs. ACE questions used the Centers for Disease Control and Prevention short ACE tool, which forms part of the US Behavioral Risk Factor Surveillance System [18]. The tool includes 11 questions on different childhood exposures to abuse and family dysfunction. These form nine distinct categories of ACE covering: physical, verbal, and sexual abuse; parental separation; exposure to domestic violence; and growing up in a household with mental illness, alcohol abuse, drug abuse, or incarceration (Table 1). The HHB outcomes examined in this study were: early sexual initiation (<16 years); unintended teenage pregnancy (<18 years); daily smoking; binge drinking; cannabis use (lifetime); heroin or crack cocaine use (lifetime); violence perpetration (past year); violence victimization (past year); incarceration; poor diet; and low levels of physical exercise (see Table 2). Although other HHBs, such as suicide attempt [11], have also been strongly linked to ACEs, our pilot survey [16] identified increased questionnaire length as detrimental to compliance, and therefore not every HHB could be included.

**Table 1:** Adverse Childhood Experiences (ACEs)

All ACE questions were preceded by the statement "While you were growing up, before the age of 18…" Responses listed are those categorized here as an ACE.		
**ACE**	**Question**	**Response**
Parental separation	Were your parents ever separated or divorced?	Yes
Domestic violence	How often did your parents or adults in your home ever slap, hit, kick, punch, or beat each other up?	Once or more than once
Physical abuse	How often did a parent or adult in your home ever hit, beat, kick, or physically hurt you in any way? This does not include gentle smacking for punishment	Once or more than once
Verbal abuse	How often did a parent or adult in your home ever swear at you, insult you, or put you down?	More than once
Sexual abuse	How often did anyone at least 5 years older than you (including adults) ever touch you sexually?	Once or more than once to any of the three questions
	How often did anyone at least 5 years older than you (including adults) try to make you touch them sexually?	
	How often did anyone at least 5 years older than you (including adults) force you to have any type of sexual intercourse (oral, anal, or vaginal)?	
Mental illness	Did you live with anyone who was depressed, mentally ill, or suicidal?	Yes
Alcohol abuse	Did you live with anyone who was a problem drinker or alcoholic?	Yes
Drug abuse	Did you live with anyone who used illegal street drugs or who abused prescription medications?	Yes
Incarceration	Did you live with anyone who served time or was sentenced to serve time in a prison or young offenders' institution?	Yes

**Table 2:** Outcome variables

**Outcome**	**Question (text in brackets is the response indicating behavior)**
Unintended teenage pregnancy	Did you ever accidentally get pregnant or accidentally get someone else pregnant before you were aged 18 years? (*Yes*)
Early sexual initiation	How old were you the first time you had sexual intercourse? *(<16 years*)
Smoking	In terms of smoking tobacco, which of the following best describes you? (*I smoke daily*)
Binge drinking	How often do you have 6 or more standard drinks on one occasion (*Weekly or daily or almost daily*)^a^
Cannabis use	How often, if ever, have you taken the following drugs…cannabis? (*any level of use*)
Heroin/crack cocaine use	How often, if ever, have you taken the following drugs… heroin/crack cocaine? (*Any level of use*)
Violence perpetration	How many times have you physically hit someone in the past 12 months? (*Any frequency*)
Violence victimization	How many times have you been physically hit in the past 12 months? (*Any frequency*)
Incarceration	How many nights have you ever spent in prison, in jail or in a police station? (*Any number of nights*)
Poor diet	On a normal day, how many portions of fruit and vegetables (excluding potatoes) would you usually eat (one portion is roughly one handful or a full piece of fruit such as an apple)? (*<2 portions*)
Low physical activity	Usually, how many days each week do you take part in at least 30 minutes of physical activity that makes you breathe quicker, like walking quickly, cycling, sports or exercise? (*<3 days*)

^a^Questions on alcohol consumption were drawn from the AUDIT C tool, and participants were provided with information on what constitutes a standard drink (UK = 10 mg of alcohol).

Questionnaires were completed by participants in their places of residence, under the instruction of a professional survey company directed by the research team. All sampled households were sent a letter providing study information and the opportunity to opt out prior to the surveyor visiting. At the door, surveyors again explained the study and its voluntary and anonymous nature, and provided a second opportunity for individuals to opt out. Participants were offered the choice of completing the questionnaire through a face-to-face interview using a hand-held computer (with sensitive questions self-completed; n = 3,852), or to self-complete using paper questionnaires (n = 158). The questionnaire took an average of 13 minutes to complete.

### Sample selection

A target sample size of 4,000 was based on ACE prevalence identified in a pilot study [16]. Sampling used a random probability approach stratified first by region (n = 10, with inner and outer London treated as two regions) and then small area deprivation in order to provide a sample representative of the English population. Samples for each region were proportionate to their population. Within each region, lower super output areas (LSOAs; geographical areas with a population mean of 1,500 [19]) were categorized into deprivation deciles based on their ranking in the 2010 Index of Multiple Deprivation (IMD; a composite measure including 38 indicators relating to economic, social, and housing issues [20]). Two LSOAs were randomly selected from each deprivation decile in each region for sampling (n = 200 LSOAs). Within each sampled LSOA, between 40 and 120 addresses were randomly selected from the Postcode Address File^®^, with 16,000 households initially sampled to allow for non-response, ineligibility, and non-compliance.

Of all sampled households, 771 (4.8%) opted out following receipt of the study letter. Household visits were made on all days of the week and between the hours of 09.30 and 20.30 hours. At least three attempted visits at differing days/times were made before an address was removed, with sampling completed once the target sample size was achieved.

Inclusion criteria for the study were: residence in a selected LSOA; age 18 to 69 years; and cognitive ability to participate in a face-to-face interview. A total of 9,852 households were visited, of which 7,773 were occupied. Of the occupied households, 2,719 (35.0%) opted out, 1,044 (13.4%) were ineligible, and 4,010 completed a study questionnaire. Thus, compliance was 59.6% across eligible occupied households visited, and 53.5% including those opting out at the letter stage.

### Statistical analysis

All analyses were undertaken using PASW Statistics v18. Only individuals with complete data relating to all ACEs, age, sex, ethnicity, and IMD quintile were included in the analysis, yielding a final sample size of 3,885. Although ethnicity was initially collected through self-identified UK Census categories, these were combined into White, Asian and Other because of the small numbers within individual ethnic groups (Table 3). Where individuals did not answer all relevant questions, adjusted sample sizes are presented in the tables.

**Table 3:** **Sample demographics and comparison with the English national population**^**a**^

	**Sample**		**Population**		***χ²***	***P***
	**n**	**%**	**n**	**%**		
**Age, years**						
18 to 29	815	21.0	8623299	24.2		
30 to 39	772	19.9	7051522	19.8		
40 to 49	795	20.5	7773559	21.8		
50 to 59	699	18.0	6426080	18.1		
60 to 69	804	20.7	5719911	16.1	72.016	<0.001
**Sex**						
Male	1749	45.0	17685329	49.7		
Female	2136	55.0	17909042	50.3	33.837	<0.001
**Ethnicity**						
White^b^	3354	86.3	30499391	85.7		
Asian^c^	308	7.9	2912044	8.2		
Other^d^	223	5.7	2182936	6.1	1.471	0.479
**Deprivation quintile**						
1^e^	782	20.1	7149675	20.1		
2	758	19.5	7305972	20.5		
3	766	19.7	7199331	20.2		
4	773	19.9	7054694	19.8		
5	806	20.7	6884699	19.3	6.423	0.170

^a^Population data obtained from Office for National Statistics, Lower Super Output Area population estimates mid-2012 [21].

^b^Including White British, White Irish, White Gypsy or Irish Traveller, White Other.

^c^Including Indian, Pakistani, Bangladeshi, Chinese, Other Asian.

^d^Including Mixed/Multiple ethnic group, Black/African/Caribbean/Black British, Other ethnic group.

^e^From 1 (least deprived) to 5 (most deprived).

Owing to highly significant correlations between all ACE types (see Additional file 1: Table S1), and consistent with ACE study methodology elsewhere [11,12], ACE counts were calculated for all individuals as a proxy for severity of childhood adversity and classified into four retrospective cohorts (0 ACEs, n = 2,084; 1 ACE, n = 881; 2–3 ACEs, n = 597; 4 + ACES, n = 323). Bivariate analyses used χ² tests with conditional binary logistic regression (LR) to examine independent relationships between ACE counts and HHBs of interest. Best-fit LR model parameters were used to calculate the numbers and proportions of each HHBs relating specifically to ACE count. Thus, for each HHB, model parameters for age, sex, ethnicity, and deprivation were applied to national and sample populations with ACE count parameters set to the observed values, and then with ACE count parameters set to zero ACEs.

## Results

The sample was not significantly different from the overall English population for either deprivation or ethnicity. However, the ACE sample had an over-representation of females and included a higher proportion of individuals in the age category 60 to 69 years, with a corresponding underrepresentation of those aged 18 to 29 years (Table 3). Individual ACEs ranged in prevalence from 3.9% with a drug-using household member during their childhood to 22.6% experiencing parental separation or divorce. After correction to national population demographics, these prevalences increased to 4.1% and 24.3% respectively. Overall, 46.4% of the sample had experienced at least one ACE (population-adjusted 47.9%; Table 4). Higher ACE counts were associated with deprivation, and were lower in Asian ethnicity populations, males, and the oldest age group. In childhood, living with a drug user, parental separation, having a household member incarcerated, and living with an alcohol abuser all increased in prevalence with deprivation and reduced with increasing age (Table 4). Experience of physical abuse, verbal abuse, or domestic violence within the childhood household was also highest in the most deprived quintiles. For all ACEs, Asian ethnicity had the lowest prevalence while ‘Other’ ethnicity had the highest prevalence for each ACE except living with a household member with mental illness or alcohol abuse. Differences by ethnicity did not reach significance for any ACE type. Variations in prevalence of ACE types by gender were significant for childhood sexual and verbal abuse and having a household member with mental illness or alcohol abuse, with the prevalence being higher in females.

**Table 4:** Bivariate relationships between participant demographics, individual ACEs and ACE count

	**Individual ACEs**									**ACE count**			
	**Parental separation**	**Childhood abuse**			**Household member**					**0**	**1**	**2 to 3**	**4+**
		**Verbal**	**Physical**	**Sexual**	**Mental illness**	**Domestic violence**	**Alcohol abuse**	**Incarceration**	**Drug abuse**										
**Prevalence, %**	22.6	17.3	14.3	6.2	12.1	12.1	9.1	4.1	3.9	53.6	22.7	15.4	8.3
**Age, years**													
*18 to 29*	34.6	17.4	11.4	4.8	13.1	10.2	10.1	6.7	6.1	47.2	25.0	19.1	8.6
*30 to3 9*	25.1	18.9	14.6	6.0	12.7	14.0	11.3	5.8	6.3	52.8	21.8	14.2	11.1
*40 to 49*	25.9	19.5	15.6	7.2	13.0	14.2	10.4	3.6	3.9	51.1	23.6	14.6	10.7
*50 to 59*	16.6	17.3	16.7	7.4	11.6	12.6	8.3	2.7	2.1	58.2	19.3	14.2	8.3
*60 to 69*	10.1	13.4	13.7	5.6	10.1	9.7	5.3	1.2	1.0	59.5	23.1	14.4	3.0
*χ²*	161.302	12.498	10.329	6.548	4.889	13.253	21.283	41.206	46.586	71.239			
*P*	<0.001	0.014	0.035	0.162	0.299	0.010	<0.001	<0.001	<0.001	<0.001			
**Sex**													
*Male*	21.4	15.8	14.9	4.5	10.0	11.5	7.9	3.7	3.8	54.3	23.8	15.0	6.9
*Female*	23.6	18.5	13.9	7.5	13.8	12.6	10.0	4.4	4.0	53.1	21.8	15.6	9.5
*χ²*	2.802	5.116	0.888	14.729	13.093	1.097	4.994	1.355	0.097	9.628			
*P*	0.094	0.024	0.346	<0.001	<0.001	0.295	0.025	0.244	0.755	0.022			
**Deprivation quintile**^**a**^													
*1*	16.8	12.7	10.4	5.1	10.6	8.3	5.2	1.4	1.8	59.1	24.9	11.6	4.3
*2*	21.8	17.2	13.6	5.3	11.5	12.8	9.1	3.3	3.4	52.5	25.1	14.8	7.7
*3*	22.5	15.5	14.2	5.2	12.9	11.2	8.4	3.0	3.1	54.2	23.1	15.9	6.8
*4*	24.3	18.4	14.9	7.6	11.3	12.3	10.1	5.8	5.4	53.8	18.9	17.3	10.0
*(most deprived) 5*	27.7	22.6	18.5	7.4	14.1	15.8	12.5	6.7	5.8	48.8	21.5	17.1	12.7
*χ²*	28.715	29.779	21.916	8.875	6.075	21.608	27.001	37.976	23.569	66.372			
*P*	<0.001	<0.001	<0.001	0.064	0.194	<0.001	<0.001	<0.001	<0.001	<0.001			
**Ethnicity**													
*White*	23.9	17.7	13.9	6.4	12.6	11.9	9.5	3.9	3.9	52.1	23.9	15.7	8.3
*Asian*	5.5	10.4	12.7	3.2	7.1	11.7	5.8	2.9	2.9	70.1	14.6	10.1	5.2
*Other*	26.5	20.2	22.9	7.2	11.2	15.2	8.1	8.1	5.8	54.7	14.8	18.4	12.1
*χ²*	56.670	12.028	14.390	5.136	8.109	2.221	4.738	10.425	2.959	49.138			
*P*	<0.001	0.002	0.001	0.077	0.017	0.329	0.094	0.005	0.228	<0.001			
**Adjusted ACE prevalence**^**b**^	24.3	18.2	14.8	6.3	12.0	13.1	9.7	4.3	4.1	52.1	22.8	16.1	9.0

*Abbreviations*: *ACE* adverse childhood experience.

^a^From 1 (least deprived) to 5 (most deprived).

^b^Adjusted to English national population by age, sex, ethnicity and deprivation quintile of residence. Sources for population data: Office for National Statistics Lower Super Output Area population estimates mid-2012, [21] and 2011 Census [22].

In bivariate analysis, the prevalence of all HHBs except low levels of physical exercise increased with ACE count (Table 5). Thus, prevalence of having had or caused unintended teenage pregnancy and all violence and criminal justice outcomes (violence perpetration, violence victimization, incarceration) was more than five times higher in those with 4+ ACEs (versus those with none). All HHBs except binge drinking were also associated with deprivation; for example, prevalence of early sexual initiation increased from 12.0% in the least deprived quintile to 22.3% in the most deprived, and prevalence of smoking increased from 12.9% to 38.1%, respectively (see Additional file 1: Table S2). After using LR models to account for the confounding effects of deprivation and other demographics, odds of all HHBs except low physical exercise were significantly higher in those with 4+ or 2 to 3 ACEs (versus none). Having one ACE (versus none) was associated with a significant increase in unintended teenage pregnancy, early sexual initiation, binge drinking, cannabis use, violence perpetration, violence victimization, and incarceration (Table 6). The impact of deprivation remained significant for unintended teenage pregnancy, early sexual initiation, smoking, binge drinking, incarceration, poor diet, and low exercise levels after accounting for relationships with ACE counts.

**Table 5:** Bivariate association between health-harming behaviors and ACE count

**Outcome**	**All**		**ACE count, %**				***χ²***_**trend**_	***P***
	**%**	**n**	**0**	**1**	**2 to 3**	**4+**		
**Sexual behavior**								
Unintended teenage pregnancy (<18 years)	5.5	3836	2.9	5.6	8.3	17.0	106.097	<0.001
Early sexual initiation (<16 years)	16.8	3374	10.0	19.4	23.0	37.8	164.629	<0.001
**Substance use**								
Smoking (current)	22.7	3885	17.7	21.8	28.3	46.4	127.022	<0.001
Binge drinking (current)	11.3	3885	9.3	13.2	12.6	16.7	18.579	<0.001
Cannabis use (lifetime)	19.5	3878	12.2	21.5	27.0	47.7	241.570	<0.001
Heroin or crack cocaine use (lifetime)	2.2	3882	0.9	1.5	4.0	9.0	84.106	<0.001
**Violence and criminal justice**								
Violence victimization (past year)	5.3	3883	2.4	4.2	10.7	16.1	137.578	<0.001
Violence perpetration (past year)	4.4	3884	2.0	3.6	8.7	13.9	119.609	<0.001
Incarceration (lifetime)	7.1	3879	3.1	8.1	10.2	24.5	182.58	<0.001
**Diet, weight and exercise**								
Poor diet (current)	15.6	3879	13.3	15.9	18.3	25.1	31.679	<0.001
Low physical exercise (current)	43.0	3881	44.1	41.4	41.2	42.7	1.434	0.231

*Abbreviations: ACE* adverse childhood experience.

**Table 6:** AORs for health risk behaviors in ACE count groups

**Outcome**	**n**	**ACE Count (reference category 0 ACEs)**							**Demographic factors**			
		***P***	**1**	***P***	**2 to 3**	**P**	**4+**	***P***	**Ethnicity**	**Age**	**Sex**	**IMD**
			**AOR (95% CI)**		**AOR (95% CI)**		**AOR (95% CI)**					
**Sexual behavior**												
Unintended teenage pregnancy (<18 years)^a^	3836	<0.001	1.95 (1.32 to 2.89)	<0.01	2.72 (1.83 to 4.04)	<0.001	5.86 (3.93 to 8.74)	<0.001	<0.05	ns	<0.001	<0.001
Early sexual initiation (<16 years)	3374	<0.001	1.93 (1.52 to 2.47)	<0.001	2.39 (1.83 to 3.10)	<0.001	4.77 (3.56 to 6.39)	<0.001	<0.001	<0.001	<0.001	<0.001
**Substance use**												
Smoking (current)	3885	<0.001	1.20 (0.98 to 1.47)	ns	1.64 (1.32 to 2.04)	<0.001	3.29 (2.54 to 4.27)	<0.001	<0.001	<0.001	<0.001	<0.001
Binge drinking (current)	3885	<0.001	1.36 (1.05 to 1.75)	<0.05	1.34 (1.00 to 1.80)	<0.05	2.08 (1.47 to 2.94)	<0.001	<0.001	<0.01	<0.001	<0.05
Cannabis use (lifetime)	3878	<0.001	1.80 (1.45 to 2.23)	<0.001	2.53 (2.01 to 3.20)	<0.001	6.20 (4.74 to 8.12)	<0.001	<0.001	<0.001	<0.001	ns
Heroin or crack cocaine use (lifetime)	3882	<0.001	1.58 (0.77 to 3.26)	ns	4.79 (2.55 to 8.97)	<0.001	10.88 (5.86 to 20.18)	<0.001	ns	<0.001	<0.001	ns
**Violence and criminal justice**												
Violence victimization (past year)	3883	<0.001	1.60 (1.04 to 2.48)	<0.05	4.42 (3.00 to 6.51)	<0.001	7.48 (4.92 to 11.38)	<0.001	ns	<0.001	<0.001	ns
Violence perpetration (past year)	3884	<0.001	1.71 (1.06 to 2.75)	<0.05	4.30 (2.80 to 6.59)	<0.001	7.71 (4.90 to 12.14)	<0.001	ns	<0.001	<0.001	ns
Incarceration (lifetime)	3879	<0.001	2.63 (1.84 to 3.77)	<0.001	3.65 (2.50 to 5.33)	<0.001	11.34 (7.67 to 16.75)	<0.001	<0.05	<0.05	<0.001	<0.001
**Diet, weight and exercise**												
Poor diet (current)	3879	<0.001	1.23 (0.99 to 1.54)	ns	1.38 (1.08 to 1.77)	<0.05	2.00 (1.49 to 2.67)	<0.001	ns	<0.01	<0.001	<0.001
Low physical exercise (current)	3881	ns							<0.05	<0.001	<0.01	<0.01

*Abbreviations*: *ACE* adverse childhood experience; *AOR* adjusted odds ratios; *IMD* Index of Multiple Deprivation; *NS* not significant.

^a^Accidentally got pregnant (females) or got someone else pregnant (males).

For each HHB, the best-fit LR models were used to calculate expected HHB prevalence in the sample and national population if no ACEs were experienced. Although causality could not be established in this study, modeling estimated that nationally 13.6% of poor diet and up to 58.7% of heroin or crack cocaine use is related to ACEs. ACEs also accounted for approximately half of all individuals experiencing violence in the past year, either as a perpetrator or a victim. At a national population level, this would account for over a million individuals being assaulted and just under 900,000 assaulting someone else at least once in the past 12 months (Table 7). Similarly, modeling suggested that nationally 37.6% of individuals who have experienced an unintended pregnancy before the age of 18 years (equivalent to 826,352 individuals) could be accounted for by ACEs.

**Table 7:** **Modeled impact of preventing ACEs at sample and national population levels on health-harming behaviors**^**a**^

	**Sample**						**Adjusted to national population**					
**Outcome**	**Current prevalence**		**Estimate with 0 ACEs**		**% change**	**Number saved**	**Current prevalence**		**Estimate with 0 ACEs**		**% change**	**Number saved**
	**%**	**n**	**%**	**n**			**%**	**n**	**%**	**n**		
**Sexual behavior**												
Unintended teenage pregnancy (<18 years)	5.4	211	3.0	117	-44.5	94	6.2	2199164	3.9	1372812	-37.6	826352
Early sexual initiation (<16 years)	14.6	566	9.4	363	-35.9	203	16.4	5821047	10.9	3870314	-33.5	1950733
**Substance use**												
Smoking (current)	22.7	880	18.7	727	-17.4	153	22.7	8075185	18.9	6742668	-16.5	1332517
Binge drinking (current)	11.3	439	9.6	371	-15.5	68	11.9	4226450	10.1	3581091	-15.3	645359
Cannabis use (lifetime)	19.5	757	12.9	500	-33.9	257	20.8	7392259	13.9	4945099	-33.1	2447160
Heroin or crack cocaine use (lifetime)	2.2	84	0.9	35	-58.3	49	2.4	861075	1.0	355251	-58.7	505823
**Violence and criminal justice**												
Violence victimization (past year)	5.3	204	2.6	100	-51.0	104	5.8	2061912	2.9	1018287	-50.6	1043626
Violence perpetration (past year)	4.4	170	2.1	81	-52.4	89	4.8	1708728	2.3	820709	-52.0	888019
Incarceration (lifetime)	7.1	276	3.3	126	-54.3	150	7.5	2683464	3.5	1259175	-53.1	1424289
**Diet, weight, and exercise**												
Poor diet (current)	15.6	606	13.5	525	-13.4	81	16.1	5712524	13.9	4933592	-13.6	778932
Low physical exercise (current)	42.9	1667	NC^b^	NC^b^								

*Abbreviations: ACE* adverse childhood experience; *NC* not calculated.

^a^See Methods section for details of modeling.

^b^Not calculated as model identified no independent impact of ACEs.

## Discussion

Results suggest that nearly half of all individuals in England are exposed to at least one adverse experience during childhood, and 9% experience four or more ACEs (Table 4). ACEs and HHBs were both associated with deprivation. Thus, four or more ACEs were reported by 4.3% of individuals in the most affluent quintile, rising to 12.7% of those in the most deprived. Equally, all HHBs, with the expected exception of binge drinking [23] increased with deprivation. However, we identified a strong relationship between ACEs and HHBs, independent of deprivation (Table 6). Modeling suggested that ACEs contributed to as many as one in six individuals smoking and one in seven with poor diet and binge drinking (Table 7). Links between such behaviors and childhood circumstances are likely to operate through the impact of ACEs on the developing brain. Thus, early life trauma can lead to structural and functional changes in the brain and its stress regulatory systems, which affect factors such as emotional regulation and fear response, and this may predispose individuals to HHBs [24]. Consequently, the impact of ACEs on engagement in anti-social behavior and problematic drug use appears particularly marked. Over half of cases of violence perpetration, violence victimization, incarceration, and heroin/crack cocaine use could be explained by ACEs. These HHBs represent major health, social, and economic burdens across communities, and when expressed in family environments mean subsequent generations are exposed to ACEs. Moreover, we found that ACEs accounted for around a third of individuals reporting early sexual initiation and unintended teenage pregnancy. Such pregnancies can mean that individuals are born into settings typically less prepared for the needs of children, with fewer resources for child-rearing, poorer parenting skills, and consequently greater opportunity for child abuse [25], again ensuring intergenerational transmission of ACEs and related harms.

Although the ACE methodology has been refined and extensively tested [26], it remains prone to issues associated with any cross-sectional study. Results rely on accurate recall and willingness to report ACEs even after assurances of anonymity. In older individuals especially, recollection of childhood issues may be limited, although studies elsewhere suggest that false-positive reports are rare [27]. Moreover, although prospective studies may allow more immediate recording of ACEs, they are ethically problematic if identification of ACEs in children does not lead to intervention [28]. Further, our measures of ACEs are in part subjective, with individuals having to self-identify childhood abuse and other stressors relating to household members (for example, mental health problems). However, despite definitional differences, independent comparable measures of ACEs for England are relatively consistent with this study. Thus, point estimates from national surveys have suggested that 5.9% of children in England live with an adult who is a dependent drinker, 2.8% with an adult who is drug-dependent, and 7.8% with an adult with a mental health problem [29]. Our estimates for exposure to these ACEs at any stage in childhood were marginally higher, at 9.7% for alcohol abuse, 4.1% for drug abuse, and 12.0% for mental illness. Our sample size (n = 3,885) and compliance (59.6% at the door and 53.5% including households withdrawing at the letter stage) were also comparable with other major national surveys (for example, British Social Attitudes Survey 2012, n = 3,248, compliance 53% [30]; Adult Psychiatric Morbidity Survey 2007, n = 7,353, compliance 57% [31]). Finally, although many individuals stated time constraints as their reason for non-participation, we could not measure whether ACEs or HHBs were of a different prevalence or displayed different relationships in non-participants.

In England and elsewhere, attempts to reduce financial and other inequities relating to NCDs have met with limited success [32]. Equally, calls to limit the promotion and sale of alcohol, unhealthy foods, and to a lesser extent, tobacco, are routinely blocked by industry [33]. However, resistance to commercial, cultural, and other environmental pressures to adopt HHBs appears to be related to childhood stressors, with nurturing, ACE-free childhoods increasing personal resilience [34]. There is a range of evidence-based interventions already available to improve parenting and support child development (for example, home visiting programs, parenting programs, social development programs). Many of these interventions have been developed and tested in North America, where they have reduced ACEs, increased child pro-social behavior, prevented HHBs, and been identified as cost-effective [17,35-37]. The evidence base is also developing for their use in the UK and elsewhere in Europe [17]. Thus, the relationship between child adversity, HHBs, and poor health and social outcomes identified here provides a compelling case for investing at scale in parental well-being, parenting skills, and coordinated health, education, and criminal justice services to prevent and identify child maltreatment. Moreover, measuring the benefits of such investments on such a multi-disciplinary basis strengthens the economic case for investment, with total savings potentially exceeding program costs within a year [38]. Critically, investing in parenting should be seen as a sustainable intervention that has the potential to break cycles of adversity, with positive parenting practices likely to be passed down through generations once established.

## Conclusions

Emerging international literature is beginning to describe consistent impacts of ACEs on behavior and both physical and mental health outcomes across a variety of nations [16,17,39]. However, empirical evidence on prevention is more limited, largely to the USA [17]. A better understanding of the potential impact of integrated, large-scale interventions is required in countries where universal health systems already support all parents and prospective parents. Thus, brief motivational parenting interventions communicating the benefits of warm and consistent parenting are largely untested, despite the success of such approaches in other areas [40]. Neurobiological studies have already identified changes to the hippocampus and prefrontal cortex associated with ACEs, while epigenetic studies are exposing gene-environment interactions with negative health consequences once exposed to stressors [24]. Consequently, a joint research agenda between epidemiological and other sciences is required to identify the points in a child’s development at which interventions to prevent ACEs are most important and when their impacts are largely immutable. Moreover, from a policy perspective, child health is typically considered to begin from conception. However, positive parenting outcomes are also impacted by spacing between siblings [41]. Further examination is required of how contraceptive and maternity services can better assist especially vulnerable parents with family planning. Moreover, although policies providing financial and other support for deprived parents are critical, their impact on decisions to conceive, reductions in child spacing, and consequently ACEs requires urgent attention [42]. Finally, measures to reduce other drivers of domestic violence and child maltreatment such as alcohol and drug use are also likely reduce childhood adversity. Although ACEs are linked with deprivation, they are by no means limited to poor communities, and consequently ACE prevention activities should be both universal and proportionate to need. Our results demonstrate that the absence of ACEs is linked with resilience to commercial and cultural pressures to binge drink, smoke, abuse drugs, adopt poor diets, engage in early and unprotected sex, and become involved in violent and criminal behavior. The importance of addressing ACEs is often hidden, along with the voices of the children affected. However, exposing the levels of ACEs experienced even in a developed country like England and investing more in their prevention makes both ethical and economic sense.

## Competing interests

The authors declare that they have no competing interests.

## Authors’ contributions

MAB designed the study, analyzed the data, and wrote the manuscript. KH contributed to study design, coordination, data analysis, and manuscript writing. NL coordinated the study, formatted data, and contributed to data analysis and manuscript editing. CP contributed to study coordination and manuscript editing. HL supported study development and edited the manuscript. All authors read and approved the final manuscript.

## Supplementary Material

Additional file 1: Table S1 Changes in odds of reporting any specific adverse childhood experience (ACE) with experiencing any other ACE. **Table S2.** Bivariate association between health-harming behaviours and deprivation quintile of residence. Click here for file
