# Supplementary material for: National household survey of adverse childhood experiences and their relationship with resilience to health-harming behaviors in England
Source: BMC Med. 2014 May 2;12:72. doi: 10.1186/1741-7015-12-72 (PMC4234527; doi:10.1186/1741-7015-12-72)
Supplement: Additional file 1: Table S1 — Changes in odds of reporting any specific adverse childhood experience (ACE) with experiencing any other ACE. Table S2. Bivariate association between health-harming behaviours and deprivation quintile of residence. [file 1741-7015-12-72-S1.docx]

**Supplementary Table 1: Changes in odds of reporting any specific ACE with experiencing any other ACE**

|  |  | **ACE** | | | | | | | | |
| --- | --- | --- | --- | --- | --- | --- | --- | --- | --- | --- |
| **ACE** | **Description** | **A** | **B** | **C** | **D** | **E** | **F** | **G** | **H** | **I** |
| **A** | Depressed, mentally ill, or suicidal household member |  | 7.88 | 6.82 | 5.41 | 2.99 | 4.30 | 4.49 | 5.34 | 5.09 |
|  |  |  | (6.20-10.01) | (4.88-9.54) | (3.87-7.56) | (2.44-3.65) | (3.43-5.39) | (3.62-5.57) | (4.34-6.56) | (3.83-6.75) |
| **B** | Problem alcohol use or alcoholic in household | 362.943 |  | 13.47 | 10.57 | 3.85 | 5.73 | 4.91 | 5.68 | 5.80 |
|  |  |  |  | (9.59-18.93) | (7.56-14.79) | (3.07-4.82) | (4.50-7.31) | (3.88-6.22) | (4.52-7.15) | (4.31-7.82) |
| **C** | Street drug use or prescription medication abuse by household member | 163.105 | 349.045 |  | 31.17 | 5.03 | 5.39 | 4.20 | 5.65 | 7.05 |
|  |  |  |  |  | (21.29-45.63) | (3.62-7.00) | (3.84-7.57) | (3.00-5.89) | (4.07-7.85) | (4.82-10.32) |
| **D** | Household member incarcerated | 119.484 | 274.666 | 644.185 |  | 5.07 | 5.73 | 3.64 | 4.61 | 5.34 |
|  |  |  |  |  |  | (3.66-7.01) | (4.11-8.00) | (2.60-5.11) | (3.33-6.38) | (3.60-7.92) |
| **E** | Parents separated or divorced | 121.288 | 154.383 | 110.756 | 115.046 |  | 3.80 | 2.64 | 3.44 | 2.55 |
|  |  |  |  |  |  |  | (3.11-4.64) | (2.19-3.20) | (2.88-4.10) | (1.95-3.34) |
| **F** | Domestic violence in household | 180.861 | 238.899 | 115.513 | 130.623 | 188.171 |  | 12.23 | 8.82 | 5.41 |
|  |  |  |  |  |  |  |  | (9.85-15.18) | (7.16-10.87) | (4.08-7.17) |
| **G** | Physical abuse | 211.589 | 202.728 | 80.266 | 63.373 | 105.728 | 693.716 |  | 15.27 | 8.48 |
|  |  |  |  |  |  |  |  |  | (12.43-18.76) | (6.45-11.15) |
| **H** | Emotional abuse | 293.490 | 263.277 | 131.268 | 100.452 | 201.327 | 528.313 | 920.458 |  | 8.61 |
|  |  |  |  |  |  |  |  |  |  | (6.54-11.32) |
| **I** | Sexual abuse | 151.363 | 164.953 | 132.949 | 85.042 | 49.137 | 166.855 | 312.176 | 315.782 |  |
|  |  |  |  |  |  |  |  |  |  |  |

Statistics shown are odds ratio (+/-95% confidence intervals) and *X*^2^ value. All relationships are significant at P<0·001.

**Supplementary Table 2: Bivariate association between health harming behaviours and deprivation quintile of residence**

|  |  | **Deprivation Quintile** | | | | |  |  |
| --- | --- | --- | --- | --- | --- | --- | --- | --- |
|  | n | **least deprived 1** | **2** | **3** | **4** | **most deprived**  **5** | X^2^ for trend | P |
| **Sexual behaviour** |  |  |  |  |  |  |  |  |
| Unintended teenage pregnancy (<18 years) | 3836 | 1.8 | 4.7 | 7.5 | 6.5 | 7.0 | 22.179 | <0.001 |
| Early sexual initiation (<16 years) | 3374 | 12.0 | 14.5 | 19.3 | 15.9 | 22.3 | 24.276 | <0.001 |
| **Substance use** |  |  |  |  |  |  |  |  |
| Smoking (current) | 3885 | 12.9 | 17.9 | 18.4 | 25.2 | 38.1 | 151.017 | <0.001 |
| Binge drinking (current) | 3885 | 10.4 | 11.9 | 14.2 | 11.8 | 8.4 | 1.306 | 0.253 |
| Cannabis use (lifetime) | 3878 | 17.1 | 20.2 | 18.0 | 21.5 | 20.9 | 4.020 | 0.045 |
| Heroin or crack cocaine use (lifetime) | 3882 | 1.9 | 1.3 | 1.7 | 3.1 | 2.7 | 4.263 | 0.039 |
| **Violence and criminal justice** |  |  |  |  |  |  |  |  |
| Violence victimisation (past year) | 3883 | 3.3 | 4.5 | 5.2 | 6.5 | 6.7 | 12.023 | 0.001 |
| Violence perpetration (past year) | 3884 | 3.1 | 4.1 | 3.8 | 5.7 | 5.2 | 8.507 | 0.011 |
| Incarceration (lifetime) | 3879 | 3.2 | 4.9 | 7.2 | 6.9 | 13.2 | 57.723 | <0.001 |
| **Diet, weight and exercise** |  |  |  |  |  |  |  |  |
| Poor diet (current) | 3879 | 9.4 | 12.7 | 13.6 | 20.5 | 21.8 | 63.537 | <0.001 |
| Low physical exercise (current) | 3881 | 39.4 | 45.5 | 39.5 | 43.3 | 47.0 | 5.614 | 0.018 |
